# Customized 3D‐Printed Resurfacing Arthroplasty for the Treatment of Severely Malunited Distal Humerus Associated With Posttraumatic Elbow Stiffness: A Case Report and Review of the Literature

**DOI:** 10.1111/os.70062

**Published:** 2025-06-02

**Authors:** Kehan Hua, Jianyu Zhang, Maoqi Gong, Yejun Zha, Xieyuan Jiang

**Affiliations:** ^1^ Department of Orthopedic Trauma Beijing Jishuitan Hospital, Capital Medical University Beijing China

**Keywords:** 3D‐printing, distal humerus, malunion, posttraumatic elbow stiffness, resurfacing arthroplasty

## Abstract

**Background:**

Posttraumatic elbow stiffness together with severe bony deformity of the distal humerus poses a great challenge for elbow surgery specialists. For young and high‐demand patients suffering from grievous bony deformity but with no/mild joint degeneration, total elbow arthroplasty is not a suitable option, and hemiarthroplasty often results in a significant amount of bone stock loss, making it difficult to perform a revision procedure. Resurfacing arthroplasty has been applied in the shoulder, hip, and knee joints, as well as the radiocapitellar joint. However, resurfacing arthroplasty for the entire distal humeral articular surface was not available among current clinical studies.

**Case Presentation:**

We report a case of a 66‐year‐old male patient who presented with elbow stiffness and grievous malunion of the distal humerus due to delayed treatment after a distal humerus fracture. However, there was only mild joint degeneration observed at the proximal ulna and radius owing to long‐term functional impairment. Total elbow replacement was not suitable considering certain inevitable manual labor he must attend. We customized a cementless and stemless resurfacing prosthesis for the distal humerus based on contralateral articular anatomy using a 3D‐printing technique with Co‐Cr‐Mo alloy. The traditional cemented stem was replaced with criss‐cross screws to provide primary stability. The bone‐implant interface was covered with a 3D‐printed trabecular‐like porous structure for bone in‐growth and several grooves designed for bone grafting, eventually forming a stable multinested mortise‐and‐tenon joint structure at the bone‐implant interface. At the last follow‐up, significant improvements in functional outcomes of the elbow joint were observed without any prosthesis‐related complications. The patient was able to return to his previous manual labor and reported improved weight‐bearing capability of the affected upper limb up to 25 kg without pain or instability. While he complained of postoperative ulnar nerve palsy, the symptom markedly improved at the final follow‐up.

**Conclusion:**

The novel prosthesis we have designed for resurfacing arthroplasty of the distal humerus can provide adequate primary stability and bone in‐growth capability, making it a reliable alternative for patients with severe bony deformity of the distal humerus and no/mild joint degeneration at the proximal ulna and radius, particularly young patients or those with high functional demands.

## Graphical abstract

We report a male patient who presented elbow stiffness and grievous malunion of the distal humerus due to delayed treatment after distal humerus fracture. A cementless and stemless resurfacing prosthesis was customized for the distal humerus based on contralateral articular anatomy using 3D‐printing technique with Co‐Cr‐Mo alloy.

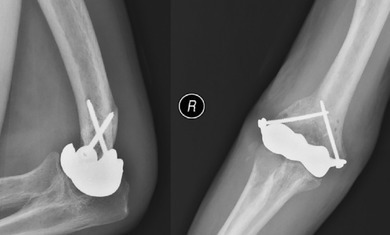

**Abbreviations**
CSRA: cementless surface replacement arthroplasty
DASH: disabilities of the arm, shoulder, and hand
HRA: hip resurfacing arthroplasty
MEPS: mayo elbow performance score
PTES: posttraumatic elbow stiffness
ROM: range of motion
TEA: total elbow arthroplasty
TKA: total knee arthroplasty
VAS: visual analogue scale

## 1 Background

Posttraumatic elbow stiffness (PTES) is a common complication after elbow trauma and surgeries [1, 2, 3, 4]. Previous studies have demonstrated satisfactory clinical outcomes concerning PTES using either open or arthroscopic‐assisted elbow arthrolysis [5, 6, 7, 8, 9, 10]. However, PTES together with severe bony deformity of the distal humerus poses a great challenge for elbow surgery specialists, with nonunion or malunion of the capitulum being the most common type [11, 12]. Under such a scenario, arthrolysis and late open reduction and internal fixation together with a hinged external fixator can be a safe and effective treatment option [11].

For patients suffering from grievous bony deformity in the trochlear region, total elbow arthroplasty (TEA) is currently the most suitable choice in clinical settings. However, for young patients or those with a high demand for upper limb function and strength, linked semiconstrained total elbow prosthesis is relatively contraindicated owing to the strict postoperative weight‐bearing restriction [13, 14, 15, 16, 17]. Thus, unlinked or convertible TEA may serve as an alternative on the basis of sufficient ligamentous reconstruction [18, 19, 20]. Nevertheless, in certain cases, there was no/mild joint degeneration at the proximal ulna and radius due to long‐term elbow dysfunction, thus making it a reasonable surgical option to perform hemiarthroplasty of the distal humerus.

Previous studies regarding elbow hemiarthroplasty have shown favorable prognosis [21, 22, 23]. However, the surgical indication is primarily for acute unreconstructable comminuted fractures, and there is limited evidence concerning its application in posttraumatic elbow disorders. Werthel et al. [24] reported poor clinical results of 16 cases with sequelae of previously treated distal humerus fractures using elbow hemiarthroplasty, including posttraumatic osteoarthritis, nonunion, ankylosis, and chronic fracture‐dislocation. The mean mayo elbow performance score (MEPS) at last follow‐up was merely 72 points. All patients exhibited varying degrees of cartilage wear, and only seven patients had no signs of loosening. There were eight complications that occurred with a reoperation rate of up to 31%. In addition, the prostheses for hemiarthroplasty commonly used usually require removing most of the distal humerus and deep reaming for the cemented stem, resulting in a significant amount of bone stock loss [22]. As such, if revision surgery becomes necessary, the insufficient residual bony structure would make the procedure more troublesome and significantly increase the risk of perioperative complications [25]. Therefore, resurfacing arthroplasty seems to be a better choice; however, there is no such prosthesis specifically designed for the entire articular surface of the distal humerus.

Based on the above, we developed a cementless and stemless prosthesis for resurfacing arthroplasty of the distal humerus, aiming to minimize bone stock removal while ensuring both primary and secondary stability. Customization using a 3D‐printing technique based on the contralateral anatomy of the distal humerus was utilized to achieve optimal joint congruency and reduce prosthetic wear. In this article, we report a case using this newly invented prosthesis.

## 2 Case Presentation

A 66‐year‐old man had suffered a terrible traffic accident 9 months before admission. He was initially diagnosed with open fractures of the distal humerus and radial head, along with a scapular fracture on the right side. Open reduction and internal fixation were performed for the scapular fracture; however, the elbow was managed by irrigation and debridement followed by immobilization for 2 months. After removing the cast, aggressive rehabilitation was carried out but provided minimal improvement. Then, he presented to our department, complaining about severely impaired range of motion (ROM) of the elbow joint, which significantly influenced his daily life activities and manual labor.

**FIGURE 1:**
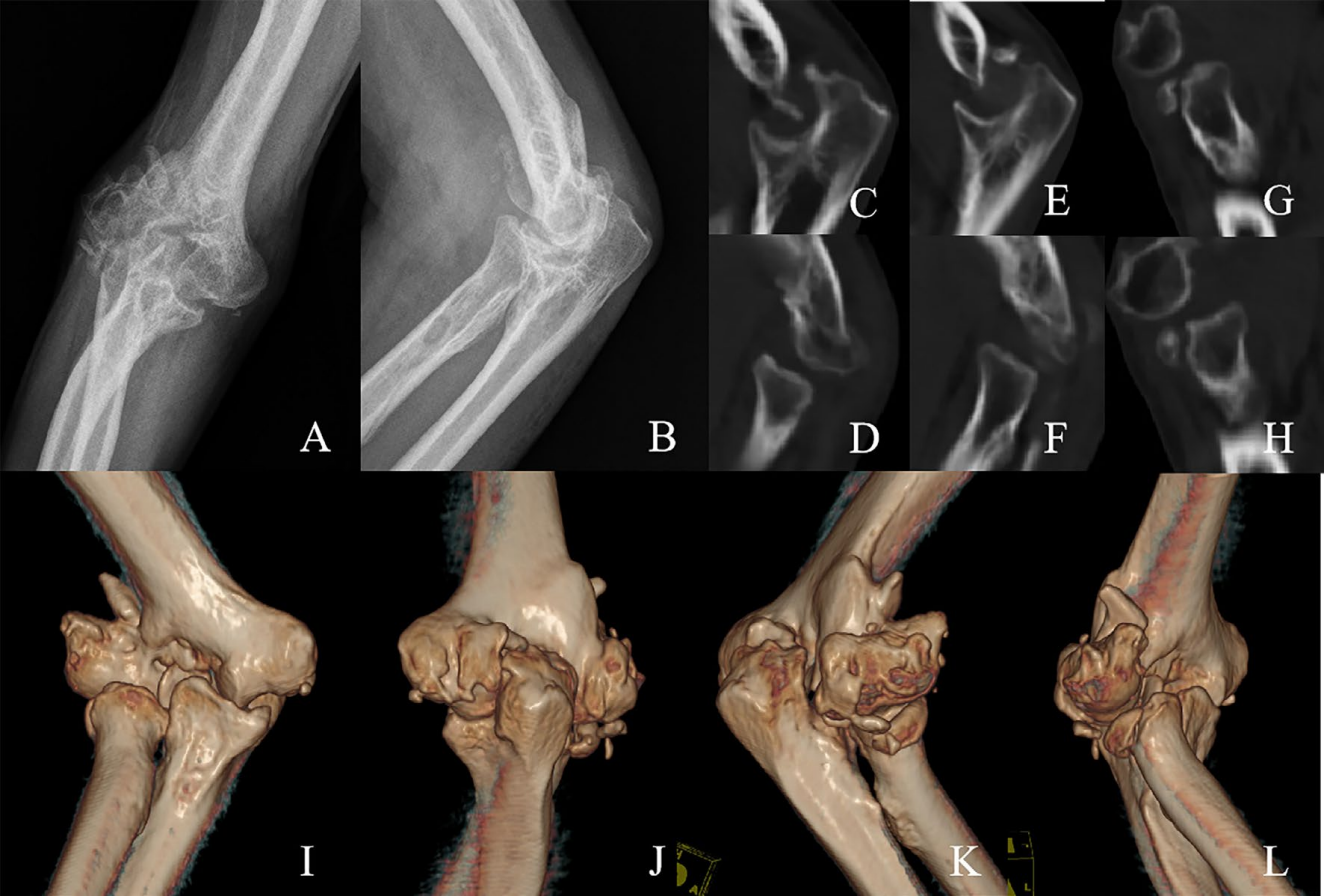
(A and B) Anteroposterior and lateral view of the injured elbow joint demonstrated bony deformity of the distal humerus. (C–F) The articular surface at the proximal ulna and radius presented with mild joint degeneration. (G and H) Nonunion of the radial head fracture with minimal displacement and articular step‐off. (I–L) 3D‐CT scan revealed grievous deformed distal humerus and heterotopic ossification.

On physical examination, the wound had healed well without adherent scar. The malunited distal humerus exhibited a palpable bony prominence on the lateral side, yet there was no tenderness around the elbow. The flexion–extension ROM measured 95‐80‐0°, while forearm pronation was 0° and supination was 26°. The neurovascular status of the right upper limb was intact. As for pain assessment, visual analogue scale (VAS) was 3 points. Functional status was evaluated using MEPS (55 points) and Disabilities of the Arm, Shoulder, and Hand (DASH) (89.17 points). X‐rays and CT scan demonstrated severe bony deformity of the distal humerus with heterotopic ossification formation and nonunion of the radial head fracture with minimal displacement and articular step‐off. For the proximal ulna and radius, the articular surface presented with mild joint degeneration (Figure 1).

In terms of treatment strategy, he was unable to adhere to the weight‐bearing restriction after TEA due to certain inevitable manual labor that he must attend. Fortunately, the articular surface of the proximal ulna and radius did not suffer serious degeneration owing to long‐term functional impairment and bone stock was relatively adequate. Therefore, after formal consent from the patient, we opted to design a customized 3D‐printed resurfacing arthroplasty prosthesis for his distal humerus.

**FIGURE 2:**
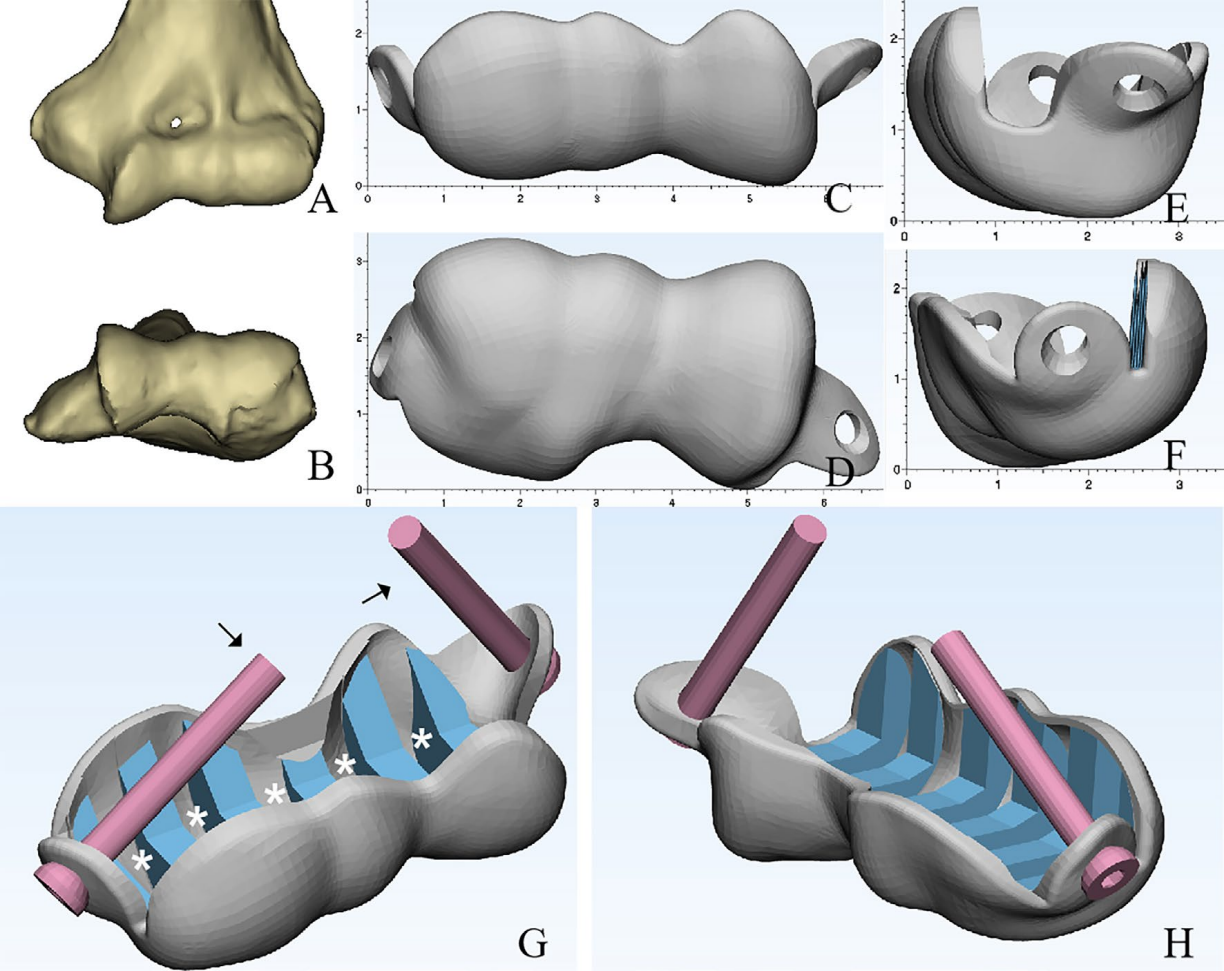
(A and B) 3D‐reconstruction of the contralateral (healthy) distal humerus. (C–F) Surface morphology of the prosthesis (based on the healthy side with mirror symmetry) and prefabricated entry portals for crisscross screws fixation. (G and H) Overall structure of the prosthesis with simulated crisscross screws (black arrows) and several grooves for bone grafting (white asterisk).

**FIGURE 3:**
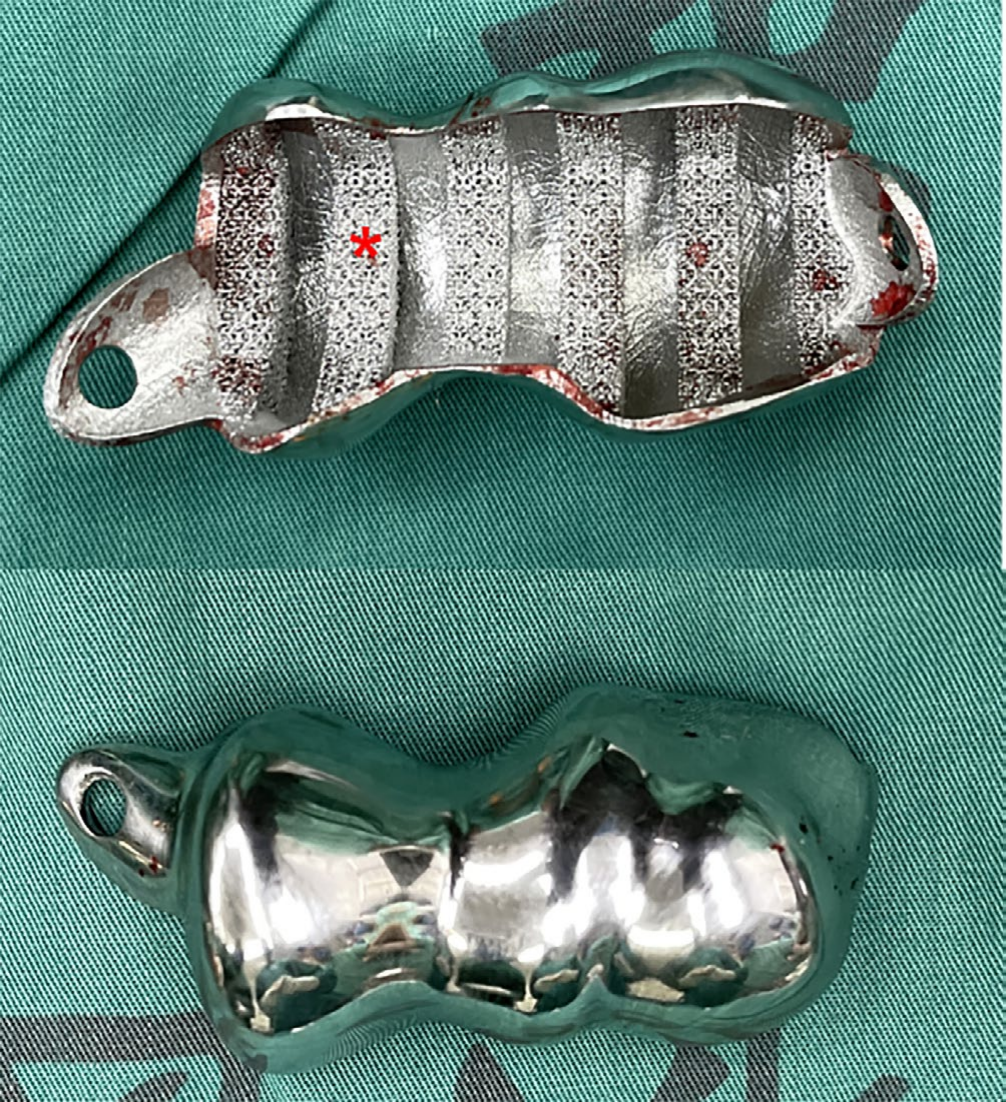
The finished prosthesis after surface polishing and sterilization (*: 3D‐printed trabecular‐like porous structure for bone in‐growth).

**FIGURE 4:**
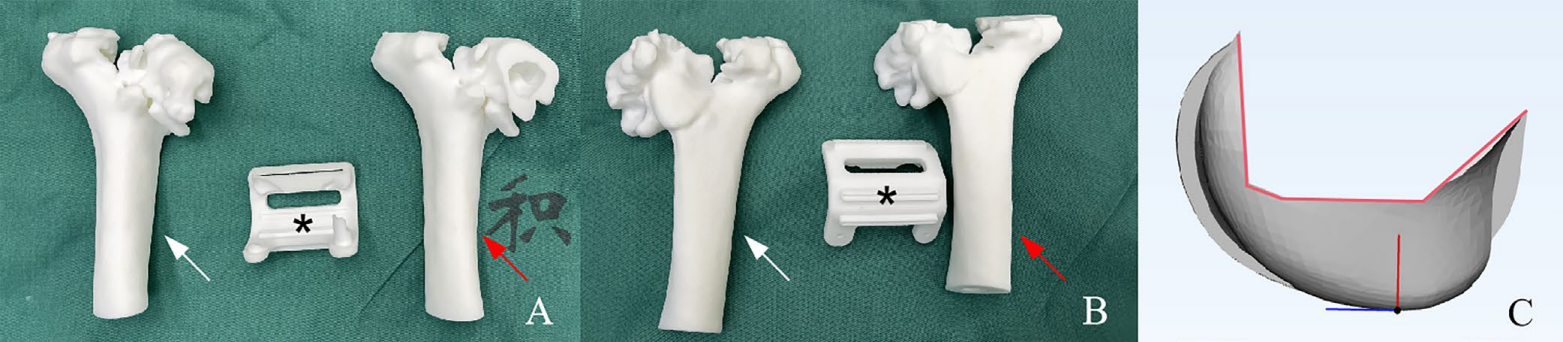
(A and B) Stepwise osteotomy was performed using a 3D‐printed customized guiding module (black asterisk). Artificial bone models were made to simulate the status before (white arrow) and after osteotomy (red arrow). (C) The contour of the osteotomy plane (red line).

CT scan of the contralateral elbow was then obtained, and the surface contour of the distal humerus below epicondylar level was used as a template to create the morphology of the prosthesis with mirror symmetry (Figure 2). For prosthesis fixation, we adopted a crisscross screws configuration instead of a traditional cemented stem, with prefabricated portals for screws insertion on the prosthesis (Figure 2). For the bone‐implant interface, we designed several grooves to facilitate bone grafting (Figure 2), and all the bone‐implant interface, except for the bottom of the grooves, was covered with a 3D‐printed trabecular‐like porous structure for bone in‐growth (Figure 3). Additionally, we 3D‐printed a customized osteotomy guiding module with several cutting planes at different angles resembling the femoral osteotomy protocol during total knee arthroplasty (TKA) (Figure 4). The prosthesis was made of Co‐Cr‐Mo alloy. After adequate surface polishing and formal sterilization, the prosthesis was ready for clinical application.

**FIGURE 5:**
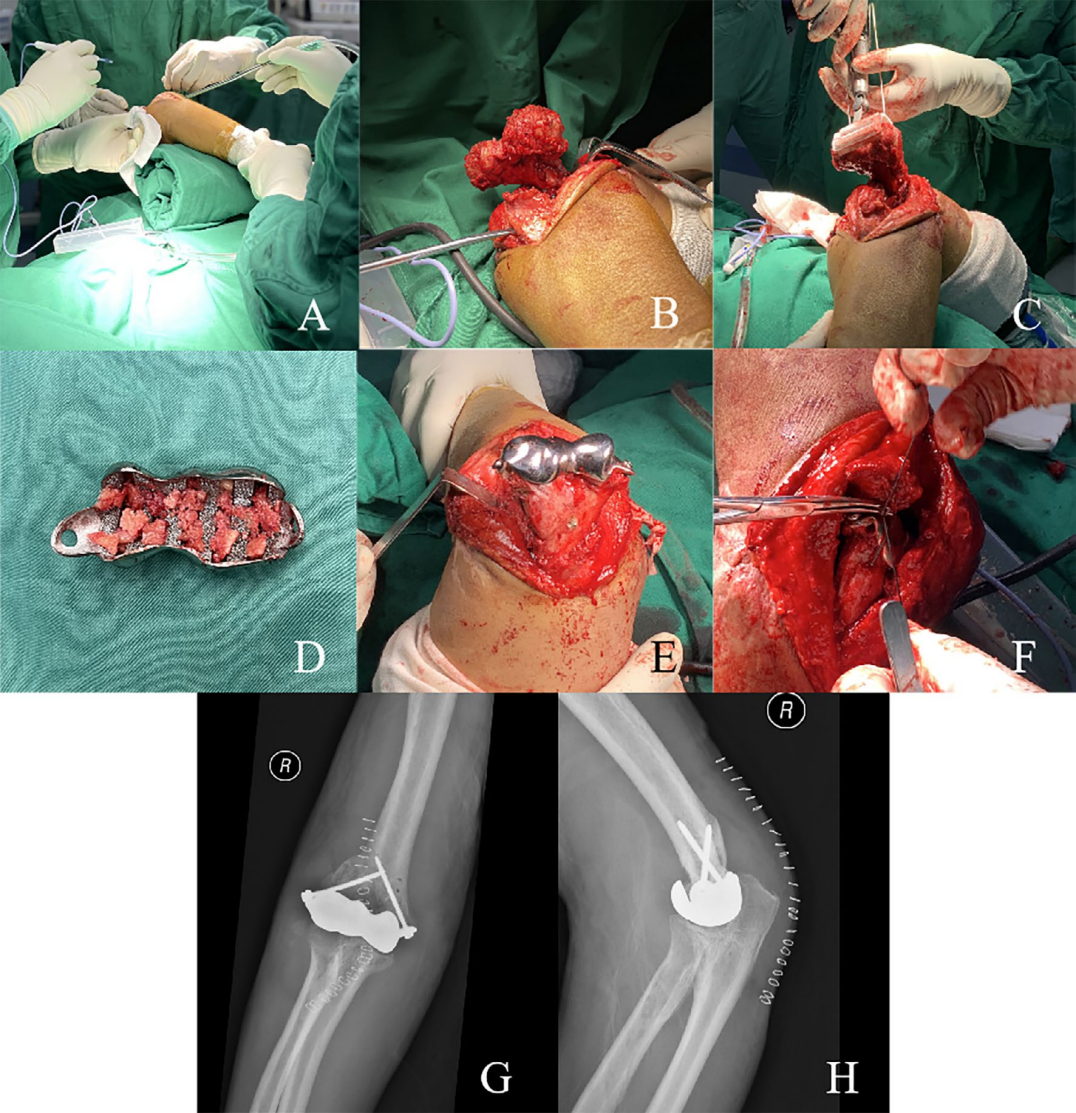
(A) The patient was prepped in a supine position and a posterior mid‐line incision was made. (B) Triceps‐sparing approach was applied and the distal humerus was dislocated through the lateral window. (C) Osteotomy was carried out with the help of the customized guiding module. (D) Bone chips harvested from the resected distal humerus were planted in the grooves. (E) The definite prosthesis was press‐fitted onto the residual distal humerus and firmly stabilized with crisscross screws. (F) Collateral ligaments were reconstructed using bone tunnels and firm sutures. (G and H) Postoperative X‐rays demonstrated successful prosthesis implantation.

The patient was administered a brachial plexus nerve block and prepped in a supine position. A posterior mid‐line incision was made, followed by exposure and protection of the ulnar nerve. Through a triceps‐sparing approach, we performed thorough arthrolysis and dislocated the distal humerus via the lateral window. After removal of any heterotopic ossification and redundant bony structure, a stepwise osteotomy was conducted using the guiding module. We then installed a testing mold, reduced the ulnohumeral joint, and achieved balanced soft tissue tension during passive ROM. Excellent joint congruency and implant position were confirmed by fluoroscopy. Before installing the definite prosthesis, the cancellous bone from the resected distal humerus was shaped into several bone chips and stuffed into the prefabricated grooves. The prosthesis was then press‐fitted onto the residual distal humerus and stabilized by two 3.5 mm fully threaded cannulated screws. A bone tunnel was created at each of the medial and lateral epicondyles using Kirschner wires, and the medial and lateral collateral ligaments were augmented by firmly suturing through these bone tunnels using 5^#^ Ethibond. The ununited radial head fragment, which was surrounded by fibrous scar tissue without separation, was left untreated. The ulnar nerve was decompressed and left in situ (Figure 5).

Within the first 2 weeks after surgery, immobilization was requested with the elbow joint in 90° flexion and the forearm in a neutral position. Then, appropriate functional rehabilitation was carried out in our department under specialized instructions from professional physical therapists, including isometric muscle contraction, gentle active, and passive ROM exercises within acceptable pain thresholds. Violent passive rehabilitation and traction by others were prohibited.

**FIGURE 6:**
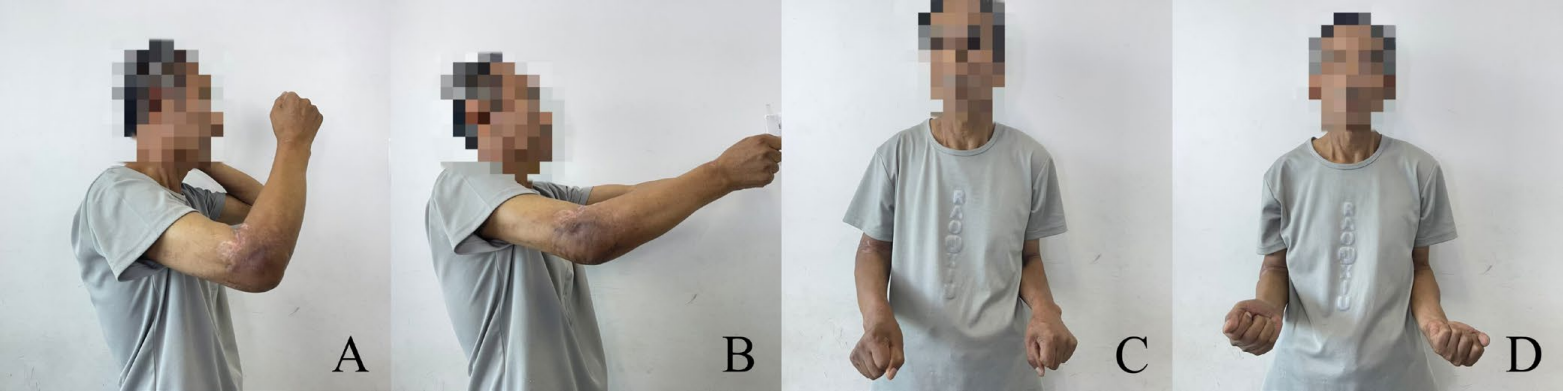
Postoperative clinical photographs of the affected elbow joint. (A–D) 1 month after index surgery.

**FIGURE 7:**
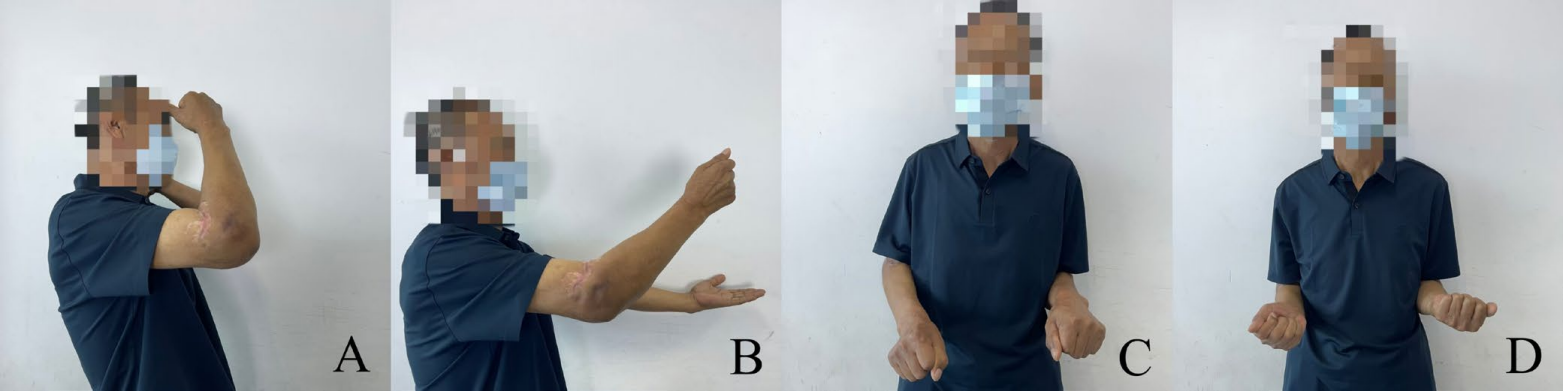
Postoperative clinical photographs of the affected elbow joint. (A–D) 2 months after index surgery.

**FIGURE 8:**
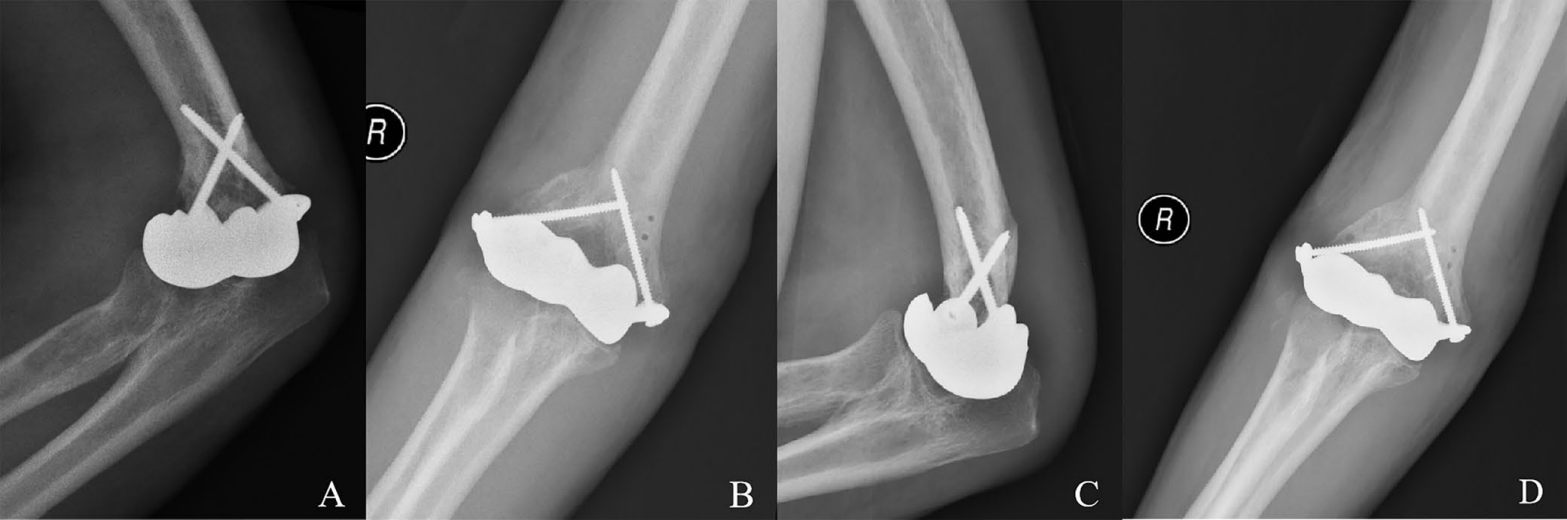
Postoperative X‐rays of the affected elbow joint. (A and B) 1 month after index surgery. (C and D) 2 months after index surgery.

Outpatient follow‐up was carried out in 1 month, 2 months, and 2 years after the index surgery, and significant improvement in elbow function has been achieved. Postoperative photographs at 1 and 2 months demonstrated a progressive improvement in the ROM of the elbow compared to the preoperative condition (Figures 6 and 7). The detailed clinical outcomes were illustrated in Table 1. Ulnar nerve neuropathy was observed postoperatively, presenting as moderate paresthesia without muscle weakness. At last follow‐up, the symptom did not completely resolve but markedly improved. No signs of infection, instability, periprosthetic fractures, or hardware irritation were observed. Regarding radiographic assessment, no signs of implant breakage, aseptic loosening, progressive cartilage wear, and heterotopic ossification were detected within 2 months after index surgery (Figure 8). Although the radiographic results at last follow‐up were not available, the patient was able to return to his previous manual labor and reported significantly improved weight‐bearing capacity of the affected upper limb up to 25 kg without pain or instability.

**TABLE 1:** Preoperative and postoperative clinical evaluation.

		Flexion (degree)	Extension (degree)	Flexion–extension ROM (degree)	Pronation (degree)	Supination (degree)	Pronation‐supination ROM (degree)	VAS	MEPS	DASH
Preoperation		95	80	15	0	26	26	3	55	89.17
postoperation	1 month	104	44	60	30	58	88	0	85	NA
	2 months	110	51	59	38	61	99	0	90	NA
	2 years	130	25	115	45	65	110	0	100	31.67

## 3 Discussion and Conclusion

### 3.1 Indications of Resurfacing Arthroplasty

TEA represents a highly mature and widely adopted technology, ideally suited for elderly patients with acute/chronic comminute distal humeral fractures or severe bony deformity associated with joint degeneration. Apparently, for patients without obvious arthritis, hemiarthroplasty of the distal humerus is more appropriate. However, current hemiarthroplasty prostheses incorporate cemented stems and require removing most of the distal humerus, resulting in substantial bone loss. If revision surgery needs to be performed, the inadequate residual bone stock and cement would make the operation significantly challenging and troublesome, making it more prone to suffer perioperative complications. Therefore, if primary bone stock is adequate, resurfacing arthroplasty seems to be a better choice under such scenarios. The prosthesis we designed is indicated for patients with severe bony deformity of the distal humerus but no/mild joint degeneration at the proximal ulna and radius, especially for young patients or those with high functional demands.

### 3.2 Previous Research on Resurfacing Arthroplasty

Resurfacing arthroplasty has been widely applied in lower limbs, such as TKA and hip resurfacing arthroplasty (HRA) for femoral head [26]. In upper limbs, a similar technique has been used in the resurfacing of the humeral head and radiocapitellar joint [25, 27, 28, 29]. Schmidutz et al. [30] quantitatively evaluated the histomorphometry of 14 cementless surface replacement arthroplasty (CSRA) implants retrieved from patients undergoing revision due to glenoid erosion. They found that the mean percentage of bone implant contact at the interface was only 20.5% ± 5.8%, which was significantly lower than that observed with cementless acetabular cups in total hip arthroplasty (29.7% [31] or 36.5% [32]). The weaker ability of bone on‐growth can be attributed to the distinct biomechanical environment between upper and lower limbs in regard to weight‐bearing. This difference determines the particularity of prosthesis design. Therefore, additional attention should be paid to resurfacing arthroplasty of the elbow joint regarding primary and secondary stability to ensure a successful outcome [33].

### 3.3 Stability Considerations and Surgical Techniques

Primary stability is mechanically achieved and relies on a firm fit and lock between the implant and bone [30, 34]. For elbow hemiarthroplasty, a substantial portion of the distal humerus is removed along with deep reaming of the medullary cavity to facilitate stem insertion and bone cement reinforcement. Although previous studies had demonstrated satisfactory primary stability and low revision rates [35], a considerable amount of bone stock loss caused by the current design would make it technically challenging if revision surgery becomes necessary. Therefore, in our case, we adopted crisscross‐type screw fixation instead of a traditional cemented stem to provide adequate primary stability, with one fully threaded cortical screw in each column through prefabricated entry portals on the prosthesis, thus forming a stable triangular structure. Park et al. [36] adopted such a configuration in the treatment of transcondylar fractures of the distal humerus, and favorable clinical outcomes have been reported. All patients in their series achieved successful bony union after 3 months. At the last follow‐up, the mean extension–flexion ROM was 12°–125° with no impairment in forearm rotation, and the mean MEPS was 93.8 points without major mechanical complications. In addition, we designed a customized osteotomy guiding module imitating the protocol of femoral osteotomy during TKA, rather than traditional en‐block resection of the distal humerus. In this way, not only can we maximize the preservation of bone stock, which can effectively enhance the holding strength of crisscross screws thereby lowering the risk of early‐stage mechanical failure, but we can also keep the prosthesis lightweight, providing greater comfort for the patient during rehabilitation or daily live activities [25]. In our case, the satisfactory short‐term outcomes without mechanical complications have proven that sufficient primary stability can be achieved by our prosthesis.

Secondary stability, also referred to as prosthetic longevity, is biologically induced and depends on sufficient bone in‐growth/on‐growth [30, 34]. Given the distinct biomechanical environment between the upper and lower limbs, a cementless elbow prosthesis solely dependent on bone on‐growth/in‐growth coating at the bone‐implant surface might not be as stable as desired. Therefore, in addition to a 3D‐printed trabecular‐like porous structure for bone in‐growth, we designed several grooves at the bone‐implant interface to facilitate bone grafting using cancellous bone of the resected distal humerus or iliac crest autografts. On the basis of adequate primary stability provided by crisscross screws fixation, successful fusion between the blood‐rich autografts and the residual distal humerus can be expected, thus forming a multinested mortise‐and‐tenon joint structure at the bone‐implant interface, which can significantly increase the contact area for bone in‐growth and render the entire construct more reliable and stable.

Furthermore, bone remodeling plays a crucial role in secondary stability [37]. Periprosthetic bone resorption can lead to aseptic loosening or mechanical failure [15]. The causes for such phenomena are still not completely understood, with bone necrosis, wear debris, and stress shielding being the most frequently discussed topics [30, 38, 39]. First, thermal damage during cement curing may compromise the viability of osteocytes, leading to periprosthetic bone necrosis [40]. In our case, a cementless design was adopted to avoid such complications. Second, wear on the polyethylene liner, which was not part of our prosthesis, may generate debris and activate resident immune cells, causing chronic inflammation and eventually osteolysis, affecting periprosthetic bone density and overall stability [41]. Finally, stress shielding has the strongest influence on the bone remodeling process [42]. Bone resorption and subsequent prosthetic loosening resulting from attenuated regional loading of the bone have been validated [43, 44, 45] For HRAs, the stem and rim serve as the main load‐bearing path, while the unloaded region beneath the implant may undergo resorption and cause femoral neck narrowing [30, 42]. For CSRAs, stress shielding is prone to occur at the prosthetic rim, leading to regional osteolysis [46, 47].

### 3.4 Complications of Resurfacing Arthroplasty and Improvements

Levy et al. [46] observed mild or progressive radiolucent lines in 30% of the humeral component and 64% of the glenoid component after CSRAs using Copeland Mark‐II prostheses, which had been attributed to the revision of two patients (1.9%). Verstraelen et al. [48] performed 33 cases of CSRAs in 27 patients using Copeland Mark‐III + prostheses and radiolucent lines were seen in six cases (18.2%), but no revision was required. Karssiens et al. [49] implanted 50 cases of Epoca resurfacing head total shoulder arthroplasty and radiolucencies were found in only two cases (3.8%) on the humeral side and three cases (5.7%) on the glenoid side; among the latter, one revision has been performed. Compared to the shoulder, the elbow joint exhibits distinct biomechanical characteristics [29]. However, the complex anatomy of the trochlea necessitates an irregular contour of the implant surface rather than a smooth hemispherical design; therefore, there is not much comparability between our implant and previous designs in terms of periprosthetic stress–strain distribution, which should be explored thoroughly by finite element analysis or bone density screening of actual patients in the future.

Cartilage wear at the native proximal ulna and radius is worth noting for such hemi‐joint replacement [21, 23]. Worse wear has been associated with higher pain levels, lower satisfaction scores, and lower quality of life [50]. Piggott et al. [35] conducted a systematic review regarding elbow hemiarthroplasty for acute distal humerus fractures. A total of 13 related articles were included, and 207 patients were enrolled. Ulnar wear was observed in 44 patients (21.8%) but only one patient eventually required revision to TEA. Schultzel et al. [51] reported the long‐term prognosis of hemiarthroplasty for the treatment of acute distal humerus fractures in 10 patients with a mean follow‐up period of 115 months. At the last follow‐up, radiography focused on the ulnar notch and radial head did not reveal any significant chondrolysis and arthrosis.

However, it is not the same for sequelae of previously treated distal humerus fractures in terms of cartilage wear comparing with acute fractures. Celli et al. [21] reviewed 17 patients who underwent elbow hemiarthroplasty using Tornier Latitude prosthesis for the treatment of chronic lesions after acute distal humerus fractures, including malunion and pseudarthrosis. Radiographic assessment detected ulnar and/or radial side wear of the articular surface in five cases (29.4%). Smith et al. [50] performed distal humeral hemiarthroplasty in five cases of malunion or nonunion. Of these cases, two were revised to TEA and the other three cases exhibited moderate ulnar wear. Werthel et al. [24] enrolled 16 patients treated by elbow hemiarthroplasty due to posttraumatic osteoarthritis, nonunion, ankylosis, and chronic fracture‐dislocation. Varying degrees of cartilage wear at the native proximal radius and/or ulna were observed in all patients with an average follow‐up of 51 months. Table 2 summarizes the key studies on severe bony deformity of distal humerus associated with elbow stiffness, highlighting their surgical strategies.

**TABLE 2:** Treatment of severe bony deformity of distal humerus associated with elbow stiffness in the literature.

Authors	Causes	Surgical treatment
Yu^11^	Malunion or nonunion of capitellum fracture	Arthrolysis by twin incisions, late open reduction and internal fixation, and a hinged external fixator
Ouyang^12^	Distal humeral nonunion	Open arthrolysis, surgical reduction, internal fixation, hinged external fixation, and selective bone grafting
Nishida^19^	Rheumatoid arthritis	Unlinked total elbow arthroplasty
Celli^21^	Malunion or pseudarthrosis of distal humerus	Elbow hemiarthroplasty
Werthel^24^	Rheumatoid arthritis	Elbow hemiarthroplasty
Watkins^29^	Elbow arthritis	Lateral resurfacing elbow arthroplasty
Smith^50^	Low distal humeral fractures with comminution and poor bone quality deemed unreconstructable	Elbow hemiarthroplasty

In order to slow down the progression of joint degeneration, we utilized the contralateral (healthy) side as a template to create the articular surface of the prosthesis using 3D‐printing technology. Therefore, superior joint congruency and minimized cartilage wear can be expected in comparison with commercially available products that feature a uniform morphology across different sizes. Our prosthesis is specially indicated for severely deformed distal humerus with no/mild joint degeneration at the proximal ulna and radius, thereby improving prosthetic longevity as much as possible. Nevertheless, long‐term clinical and radiographic outcomes in more patients are needed to confirm the effectiveness and reliability of our design. Additional research on material modification, fixation configuration, and surface coating of the prosthesis is required to optimize its performance.

## 4 Conclusion

The novel prosthesis we have designed for resurfacing arthroplasty of the distal humerus can provide sufficient primary stability and bone in‐growth capability. It can serve as a reliable alternative for patients with severe bony deformity of the distal humerus but no/mild joint degeneration at the proximal ulna and radius, especially for young patients or those with high functional demands. However, further research is required to refine the material, fixation configuration, and surface coating of the prosthesis. Also, larger prospective cohorts with long‐term follow‐up are needed to optimize the clinical performance of our prosthesis.

## Author Contributions

K.H. and J.Z. collected the patient's baseline information and imaging data. Y.Z., M.G., and X.J. performed the surgery. K.H. and J.Z. collected the follow‐up data. K.H. and J.Z. are major contributors in writing the manuscript. All authors were major contributors in the revision of this manuscript. K.H. and J.Z. contribute equally to this manuscript. Y.Z. and X.J. are both corresponding authors. All authors read and approved the final manuscript.

## Ethics Statement

This study had been approved by the Institutional Review Board (IRB) of Beijing Jishuitan Hospital, Capital Medical University (JST‐K2024‐031‐00).

## Consent

We confirmed that the written consent to publish this manuscript was obtained from the study participant.

## Conflicts of Interest

The authors declare no conflicts of interest.

## Data Availability

The datasets used and analyzed during the current study are available from the corresponding author on reasonable request.
